# Diseases of the Anus and Rectum

**Published:** 1902-06

**Authors:** 


**Diseases of the Anus and Rectum.** By D. H. GOODSALL and W. E. MILES. Part I. Pp. 311. Longmans, Green, and Co. 1900.

This is an excellent book based upon the work and mode of practice of the authors. The illustrations are numerous and the photographic reproductions good. The anatomy of this important region is well described. The method of defining the anal region by quadrants is to be commended, and we observe that the authors prefer to use the left forefinger for examinations. Operation for tuberculous fistula is favourably spoken of. The method of arrest of hemorrhage after pile operations, by the insertion of a tube with packing around, is said to have never failed. The claim of the writers to a recent modification of Salmon's operation (p. 292) is unjustifiable; for it has been in use for the last fifteen years, and probably for a much longer time.

